# Key informant perspectives of suicide prevention in Australia

**DOI:** 10.1186/s12889-024-20943-6

**Published:** 2024-12-18

**Authors:** Bridget Bassilios, David Dunt, Karolina Krysinska, Anna Machlin, Danielle Newton, Dianne Currier

**Affiliations:** Centre for Mental Health and Community Wellbeing, School of Population and Global Health, University of Melbourne, Melbourne, Australia

**Keywords:** Suicide prevention, Suicide prevention policy, National suicide prevention strategy, Stakeholder perspectives

## Abstract

**Background:**

Suicide prevention policy in Australia is in a period of reform. The National Suicide Prevention Office is leading the development of a new National Suicide Prevention Strategy (Strategy). Stakeholder input is a critical element in the development of the new Strategy. This article describes key informant views about government-led suicide prevention efforts in Australia obtained as part of an environmental scan conducted as one input to inform the Strategy development process.

**Methods:**

We interviewed 24 key informants in November and December 2022. Key informants were purposively recruited to ensure representation from cross-jurisdiction government departments/agencies, peak bodies and leaders in the suicide prevention sector, people with lived experience of suicide, and suicide prevention researchers. We enquired about successes, challenges, and opportunities. NVivo was used to conduct thematic analysis.

**Results:**

Key themes identified as successes in Australia’s suicide prevention efforts included: leadership and funding for programs, services, and research; valuing the collective lived experience voice; moving towards a whole-of-government/system approach; and high community and political suicide (prevention) awareness. Key themes emerging as challenges in the sector were: defining the suicide prevention sector, limitations in the service system, workforce issues, and building the evidence base. Key themes mentioned as opportunities for improving suicide prevention efforts were: leveraging the current unprecedented awareness and desire for collaboration among multiple stakeholder groups; adopting wellness rather than crisis-driven models of care; including lived experience and co-design in all stages and aspects of policy planning, service development, and evaluation; and investing in data, research, and evaluation.

**Conclusions:**

Key informants from across the suicide prevention sector in Australia identified a range of issues for consideration in the development of Australia’s new National Suicide Prevention Strategy which are also relevant for suicide prevention policy and program development in other high-income countries. Key issues include the need for concerted efforts to define and build the capacity of the suicide prevention sector, implement and monitor a whole-of-government approach that includes wellness models of care and lived experience, and bolster the evidence base. These efforts require effective leadership and resourcing.

## Background

Suicide is a major public health concern. Globally, around 703,000 people die by suicide each year, with families and communities grieving their loss [1]. Over the past decade, the age-standardised suicide rate in Australia has increased by around 10% from 11.2 in 2013 to 12.3 per 100,000 (equivalent to 3,249) people in 2022 and suicide was the 15th leading cause of death [2]. Figures are even higher for people who attempt suicide. This rate increase is partly attributable to improvements in data accuracy and quality over time [3]. However, despite investment in suicide prevention strategies, services and programs, Australia’s suicide rate does not appear to be decreasing, with its 2019 rate sitting in the middle of other OECD and G20 countries (18 of 36 and 23 of 43, respectively) [4].

Evidence from systematic reviews demonstrate that certain types of suicide prevention interventions can be effective [5, 6]. Examples of interventions that work to differing extents include training primary care physicians to detect and treat depression, active outreach post-discharge from a psychiatric facility or a suicidal crisis, and means restriction [6]. Different interventions are appropriate for preventing deaths by suicide and suicide attempts, and multilevel interventions (having components delivered in different healthcare settings and by different providers) are more effective than single level interventions [5]. Additionally, recent analysis has shown that individual components of national suicide prevention strategies are not associated with changes in the suicide morality rate in 29 lower middle-income and high-income countries [7]. The most common individual components in those strategies were education and training (e.g., gatekeeper training and training of primary care physicians; 96.5%), surveillance (93.1%) and oversight and coordination (79.3%); and least common were psychotherapy (20.6%) and crisis intervention (37.9%) [7]. Given the limited evidence for effectiveness of many of the specific components of suicide prevention strategies, there has recently been increasing interest in a public health approach, which considers multi-layered systems-based interventions.

A public health approach acknowledges the complex interplay of biological, psychological, clinical, social, and environmental factors that contribute to suicidal behaviour [8]. This is supported by review findings which show that addressing social determinant risk factors (e.g., economic recession, unemployment, personal financial problems) using universal interventions such as unemployment benefits, employment protection legislation, higher minimum wage and active labour market programs may reduce suicide at the population level [9]. Importantly, the public health approach includes interventions that address social determinants and protective factors as well as individual risk factors [10]. For example, a recent study examining current priorities in Australian suicide prevention research based on trends in funding, publications, and stakeholder priorities reported a transition away from suicide to suicide attempts, and future suicide prevention research priorities including suicide attempts, protective factors, social determinants, community settings, and interventions, and a focus on strengthening effective research translation into practice [11].

Effective national-level suicide prevention strategies are needed to address the scale and complexity of suicidal behaviour [12]. In Australia, the National Suicide Prevention Office (NSPO) was established in 2022 [13] with a priority task to lead the development of a new National Suicide Prevention Strategy (Strategy), which reflects contemporary evidence-based and evidence-informed suicide prevention efforts. The new Strategy is a key component of the work being led by the NSPO to drive a nationally consistent and integrated approach to suicide prevention.

This study describes policy and peak body leader (key informant) perspectives of successes, challenges and opportunities in Australia’s suicide prevention efforts. The study was part of an environmental scan of the government-led suicide prevention system in Australia conducted as one input to inform the Strategy development process. Details of the other components of the environmental scan are described elsewhere [14].

## Method

We conducted interviews with key informants from across the government-led Australian suicide prevention sector to elicit their views on government-led suicide prevention efforts in Australia. Our method describes key characteristics identified in the consolidated criteria for reporting qualitative research (COREQ) [15]. These study characteristics comprise three domains: research team and reflexivity, study design, and data analysis and reporting.

### Key informants

We used our networks and worked with the NSPO to identify representative key informants whose views would supplement the broader environmental scan of government-led suicide prevention activity [14]. Noting the primary purpose of consultations with key informants was to ensure we had included all relevant government-led suicide prevention activity in our environmental scan and the secondary purpose was to elicit their views on related successes, challenges and opportunities, a sample size was not predetermined but we undertook purposive recruitment until sampling frame requirements were met for quality and sufficiency of information [16]. Therefore, we approached key policy makers (national, state and territory), researchers, and lived experience and peak body leaders involved in government-led suicide prevention efforts to participate in this study.

### Procedure

All key informants were given a plain language statement and provided informed verbal consent to being interviewed and for interviews to be audio-recorded and transcribed. This included consent for findings to be used to inform the development of the Strategy and to be published in academic journals and/or presented at scientific conferences. DD, KK, BB and AM conducted the interviews. Individual or group interviews were semi-structured consultations conducted by phone or Zoom in November and December 2022.

We provided key informants with relevant contextual information regarding the NSPO’s remit and asked them about the nature and extent of suicide prevention activity in their jurisdiction or sector with a focus on successes, challenges, and opportunities. This study summarises findings from three interview questions:

1. What are the key successes in relation to your department’s/agency’s suicide prevention approach?
2. What are the key challenges in relation to your department’s/agency’s suicide prevention approach?
3. What are the key opportunities in relation to your department’s/agency’s suicide prevention approach?

Additional questions related to identifying relevant documents and information for the overall environmental scan, the findings from which are described elsewhere [14].

The individual and group consultations were audio-recorded and summarised, and automated transcripts were generated for interviews conducted by Zoom.

We obtained approval to conduct consultations with key informants from The University of Melbourne’s Human Research Ethics Committee (ID 25279).

### Data analysis

DN conducted the analysis of content and themes from the interviews in NVivo 12 using a theoretical semantic analysis approach, guided by our theoretical interest in the topic and the explicit surface meanings of the data [17]. Initially, an a priori coding framework was based around the interview questions. Based on the content of interview transcripts and summaries, an inductive approach was then utilised to refine the framework to capture the full range of responses and to organise them thematically. BB read and independently coded the transcripts and summaries to ensure consistency of theme coding. No inconsistencies in theme coding were identified.

## Findings

### Key informants

Table 1 shows that 24 key informants participated in 14 interviews and represented the following groups: (1) Australian and state/territory health government departments (*n* = 17); (2) lived experience of suicide and priority group peak bodies (*n* = 4); (3) suicide prevention research and education leaders (*n* = 2); and (4) a mental health at work service provider (*n* = 1). Most key informants were senior executive advisors and managers who were represented in all 14 interviews. All but one of the invited key informants took part in an interview, with the one who did not participate (representing a group of key service provider agencies) directing us to relevant information that we included in the broader environmental scan [14].

Consistent with the views of some qualitative researchers, we did not quantify the frequency of themes to minimise the risk of undermining the legitimacy of any insights derived from a relatively small number of interviews [18].

**Table 1:** Agencies represented by number of key informants

Departments/agencies	*n*
**Government**	
Australian Government Department of Health and Aged Care – Suicide Prevention and Digital Branch, and Suicide Prevention Section	2
Australian Capital Territory Mental Health and Suicide Prevention Division	3
New South Wales Mental Health Commission	2
Northern Territory Health	1
South Australia Office of the Chief Psychiatrist	4
Queensland Office of Chief Psychiatrist	1
Tasmanian Department of Health	1
Western Australian Mental Health Commission	1
Victorian Department of Health, Suicide Prevention and Response Office	2
**Peak bodies**	
Roses in the Ocean	2
LGBTIQ + Health Australia	2
**Research and education leaders**	
LIFEWAYS - Suicide Prevention Research Leadership and Translation	1
Australian Institute for Suicide Research and Prevention	1
**Provider of Australia-wide mental health at work services**	
MH@Work	1
**Total**	**24**

### Successes

Themes identified by key informants as successes in Australia’s suicide prevention efforts were: (1) leadership and funding for programs, services, and research; (2) valuing the collective lived experience voice; (3) shifting to a whole-of-government/system approach; (4) high community and political suicide (prevention) awareness; and (5) other successes.

#### Leadership and funding for programs, services, and research

Key informants identified leadership and funding for suicide prevention as key successes in Australian government-funded suicide prevention activity. They mentioned national government infrastructure enables suicide prevention efforts to occur. For example, they noted substantial funds have been made available and continue to increase, which means peak bodies (e.g., Suicide Prevention Australia and Roses in the Ocean providing sector and lived experience leadership, respectively) provide the foundation for suicide prevention efforts as illustrated by this quote:

A lot of the things we fund and are involved in provide the infrastructure for a suicide prevention sector in Australia. There are a lot of things like peak bodies, the training we fund, the networks we’ve established … are the backbone of the sector that enables a lot of the other initiatives to run the way that they do.

Key informants also spoke about the success of national government and National Aboriginal Community Controlled Health Organisation (NACCHO) leadership in First Nations peoples’ social and emotional wellbeing. Within the theme of leadership and funding, key informants mentioned government investment in various suicide prevention programs, approaches and research as a success. Selected examples are described in Box 1.

**Box 1:** Examples of Australian government investment in suicide prevention programs and approaches

• The National Suicide Prevention Leadership and Support Program (NSPLSP) including among others, programs providing media training (developed and implemented under the auspices of Everymind and Mindframe) [19, 20]. Under the NSPLSP, the Australian Government funds 40 projects as a mechanism for providing essential sector leadership, reform, advocacy, research and translation, and services targeting people who are disproportionately impacted by suicide.
• Nationally funded research that is integral for identifying where and how to invest in suicide prevention efforts. For example, LIFWAYS, funded under the NSPLSP, as a leader in suicide prevention research [21]. They are fourth in the world in terms of their publication output, so the organisation is “punching above their weight internationally”.
• The digital mental health program, which is considered vital for increasing overall access to services.
• The Way Back Support Service [22] (aftercare) for individuals and their families following a suicide attempt.
• The local suicide prevention networks in New South Wales.
• Implementation of the Zero Suicide in healthcare model (to better support people in crisis presenting to emergency departments) by the Gold Coast, which led to other hospitals and health services in Queensland implementing this program [23].
• The Connecting with People program in Tasmania, which aims to support a compassionate approach to suicide prevention [24].
• Introducing the Mental Health and Wellbeing Promotion Office in Victoria [25] was viewed as a “big first step in a whole of systems approach, signalling investment and legitimising status”.
• Current refinement of a postvention support approach in South Australia.
• The establishment of a suicide register in South Australia in 2021, data from which is used to build community support for prevention activities.
• Well-developed Indigenous programs in Western Australia.
• Well implemented program logic models in Western Australia, which are imperative for designing effective interventions.

#### Valuing the collective lived experience voice

Bringing the collective voice of lived experience into the field was also highlighted as a key success. Key informants articulated that they witnessed this in the form of more input from those with lived experience into models of care, and increased recognition of the importance of engaging diverse organisations representing different groups with lived experience (i.e., LGBTIQ+, First Nations) in co-design. This is exemplified by one key informant commenting:

Lived experience has been enshrined into policy since 1992, when the Australian national mental health ministers came together and recognised … they called it consumer participation, is central to policy … from the Royal Commission findings that Victoria has now undertaken, in the last two years, 65 recommendations of which lived experience and co-production are entrenched.

#### Shifting to a whole-of-government/system approach

Two successes that were illustrative of a whole-of-government/system approach were described. One involved the Western Australia Health Department co-commissioning programs and services with other portfolios such as the Departments of Housing and Education. The other involved Northern Territory establishing cross-domain policy through cross-domain working groups, which is facilitated by being a small jurisdiction and has been particularly successful in bringing people together to move towards a whole-of-system approach:

We’ve had a whole-of-government suicide prevention governance committee here … since ~ 2007… recognised as an innovative model…because we’re a small jurisdiction, we can have a whole-of-government … committee made up of 17 departments, agencies, peak bodies, Primary Health Networks (PHNs), Commonwealth and State Departments etc. that sit down quarterly…about suicide prevention…Something to share…with the nation.

#### High community and political suicide (prevention) awareness

High community and political awareness about suicide and its prevention including stigma reduction and recognition of diverse social determinants was cited as another achievement, evidenced by “more conversations now about suicide prevention than ever before”.

#### Other successes

Two additional successes mentioned were the continued sustainability of postgraduate programs in suicide prevention studies and the establishment of the Suicide Prevention Council in South Australia, tasked with preparing and maintaining the South Australian Suicide Prevention Plan and recommending suicide prevention and postvention policies and programs to the Minister for Health and Wellbeing.

### Challenges

Key informants identified challenges in the suicide prevention sector requiring additional government effort grouped across five key themes: (1) defining the suicide prevention sector; (2) limitations in the service system; (3) workforce issues; (4) building the evidence base; and (5) other challenges.

#### Defining the suicide prevention sector

The first theme among challenges focused on defining the suicide prevention sector, with one key informant stating:

No one has really defined what actually counts as suicide prevention versus other activities. So, when we say that we all need to work together, we’re not always sure who really needs to be around the table because we haven’t actually come to an agreed position … I think it could actually create a lot of clarity and a lot of stronger engagement particularly in the areas that have traditionally been neglected or not invited to the table.

This problem was evident in tensions in key informant views of suicide prevention in relation to mental health as demonstrated by the following quotes:

One of the other challenges we have from a systems level is the mistaken assumption that suicide prevention is just part of the mental system…but suicide prevention activities are distinct from … and shouldn’t be inflated with mental health;

and

My concern is we have two movements now. We have the mental health lived experience movement and the suicide prevention lived experience movement, and I think that’s to our detriment because we can’t keep separating suicide from mental health.

#### Limitations in the service system

The second group of challenges relate to various characteristics, approaches, or limitations of the service delivery system. Specifically, key informants mentioned limited services including limited community-controlled organisations in certain jurisdictions, and limited resources and/or funding, all of which were considered to hamper innovation. It was also noted that new initiatives (e.g., Safe Havens [26, 27] involving lived experience and peer-led service provision) need longer term funding to facilitate staff retention and implementation at more sites. Key informants also identified challenges associated with prevention and early intervention response timeliness in terms of ability to respond quickly to emerging risks of suicide or to intervene before a person becomes suicidal as exemplified by this quote:

Being able to respond quickly to emerging risks of suicide and invest more in upstream, understand where we need to focus efforts about our resources into the earlier prevention and intervention is an ongoing challenge.

Key informants spoke about system integration as a challenge, which they thought could be addressed through warm handovers, facilitated through aftercare services (e.g., The Way Back Support Service) that can fund clinical coordination.

Further issues within service system included the complexities associated with obtaining input from community groups and agencies and major policy plans excluding consideration of First Nations peoples. Sharing service information with the public, providers and other services was identified as challenging, with the suggestion that this could be at least partially overcome through leveraging scalable, low-cost services/initiatives such as Mental Health First Aid (early-intervention training programs that equip people to recognise and respond to someone experiencing a mental health problem) [28]. Key informants also noted that it is difficult to find new local programs, necessitating adaptation of overseas programs, which requires executives and providers working together.

#### Workforce issues

Workforce issues were the third category of challenges mentioned. This included issues such as: lack of workforce including an exhausted existing workforce due to COVID-19 (exacerbated by the related closure of national borders and the effects of fires and floods); sustaining people in the workforce and ensuring the workforce is motivated to engage in suicide prevention for safety, rather than litigation, reasons. The challenge of effectively integrating non-clinical and clinical workforces so that people who require clinical services can have those needs met was also noted:

It’s great to have a peer lived experience workforce, it’s great to have a non-clinical workforce with increased capability but … it’s like the horse has bolted … the needs of people who really need clinical services are going to be unmet.

Key informants reflected that this integration challenge is exacerbated by ongoing opposition to, and limited recognition of, the value of lived experience leadership and peer-led services in suicide prevention by government and the sector, projects not continuing beyond the pilot phase, and lack of ongoing funding for programs. Simultaneously, it was acknowledged lived experience is not sufficient to qualify as part of the suicide prevention workforce: “We need to be careful that we don’t make assumptions that everyone with lived experience wants to work in this space or has the appropriate knowledge and skills”.

Although there was recognition of the value of Roses in the Ocean as a national suicide lived experience organisation, it was not considered suitable for engaging First Nations people who do not use the term lived experience. Another view was that Roses in the Ocean is perhaps “too small and overstretched for the role it seeks”.

#### Building the evidence base

Fourthly, challenges were identified associated with building the evidence including measuring outcomes of suicide prevention efforts in a way that is meaningful and ensuring good cross-linkage data and surveillance while still protecting the data. Additionally, it was noted that to do so, takes time: “Research takes time to work out what kind of intervention is working well and for whom, and it takes time to get the evidence up to speed”.

This is exacerbated by publication lag which often results in papers not being published for up to a year after they have been submitted to a journal. COVID-19 has also meant that opportunities to present research findings at conferences have been reduced.

#### Other challenges

Other challenges were working relationships between national and state/territory governments and being perceived as a political entity rather than one making data-based decisions. The latter challenge was mentioned by a key informant from an agency representing a specific focus population.

### Opportunities

Opportunities for improving suicide prevention efforts were categorised into themes including: (1) leveraging the current unprecedented awareness and desire for collaboration among multiple stakeholder groups; (2) adopting wellness rather than crisis-driven models of care; (3) including lived experience and co-design in all stages and aspects of policy planning, service development and evaluation; (4) investing in data, research, and evaluation; and (5) other opportunities.

#### Leveraging unprecedented awareness and desire for collaboration among multiple stakeholder groups

Key informants noted that the time is right to improve suicide prevention efforts in Australia and to involve a more diverse range of stakeholders in these efforts. They noted an unprecedented strong current national government focus on suicide prevention over the past two-to-three years, dedication of researchers, improved community awareness of mental health and suicide prevention issues and needs, and a genuine desire for collaboration by diverse stakeholder groups. One informant commented:

We’ve never had more collaboration …or acknowledgement of suicide prevention as a standalone discipline …now that we’ve reached this position of everyone genuinely wanting to work together and not the competitive environments that we’ve had in the past …we have the opportunity to really … cater our services to the very diverse and complexity of people that we serve in Australia and hopefully really start to move the suicide prevention rate in the right direction.

Opportunities to improve collaboration for suicide prevention that were identified include: partnerships between Commonwealth and States and Territories under the National Mental Health and Suicide Prevention Agreement, leveraging the existing good relationships between state government and PHNs, engaging with people with lived experience and tailoring services directly to their needs, and building on cross-domain policy through cross-domain working groups.

#### Adopting wellness rather than crisis-driven models of care

Moving away from crisis driven, reactive approaches to wellness models of care was another opportunity mentioned by key informants. They elaborated that one way this could be achieved is through “early intervention to take a much more universal approach … early childhood attachment, facilitating increased coping and resilience … build coping strategies”.

Other options mentioned include establishing an Indigenous Health and Wellness Centre, further involving local government and the community sector, and developing additional place-based priority groups initiatives (e.g., by age group or targeting groups such as international students, LGBTIQ+, and veterans etc.) to increase levels of engagement, and reduce loneliness and social isolation.

#### Including lived experience and co-design in all stages and aspects of policy planning, service development and evaluation

The importance of including lived experience and co-design in all aspects of suicide prevention was highlighted as another key opportunity as illustrated by these quotes:

…that lived experience lens be across whole of government in all those areas that we’ve talked about, so that we have lived experience of people who have been homeless working in the portfolio of housing;

and

…and that we are to be leaders, and to be part of every step of the reform process, the implementation process, delivery, and then evaluation under the co-production methodology.

One mechanism mentioned for achieving this is through better recognition of the organisations representing people with lived experience and their ability to act in an advisory capacity in decision making. A key informant representing one focus population mentioned their own advisory role with the NSPO as positive. There was also recognition of associated hesitancy of peer-based approaches due to limited data and the data that is available does not demonstrate positive outcomes on suicide and suicide attempt rates. However, key informants reported that current programs and models are building greater confidence in peer-based approaches and noted that the peer workforce needs to be provided with more support and training. They also highlighted the value of having government/peak body leaders who understand suicide prevention in focus populations (e.g., Indigenous, LGBTIQ+, CALD communities) to foster compassion and understanding.

#### Investing in data, research, and evaluation

Some opportunities identified related to data, research, and evaluation. Specifically, it was suggested that opportunities be explored for cross linkage surveillance data with universities and other stakeholders:

There’s need for much more open cross data linkage with proper monitoring around the use of the data… We have the resources, we’re a wealthy country, we’re a lucky country…to look at all the different pathways to care and the sorts of characteristics of groups… experiencing suicidality.

Key informants also stated that a commitment to evaluation is desirable. Additionally, one key informant mentioned that the launch of LIFEWAYS’ new work program focusing on research translation is a significant opportunity to ensure that their research effort is utilised by relevant stakeholders:

New work program … now … focusing on research translation… I think there’s just a really great opportunity there to sort of make sure that … translates into policy and practice … there’s about 70 to 100 publications coming out each year here in Australia on suicide prevention specifically … So, where does it all go is the question. We want to make sure that’s being utilised.

#### Other opportunities

Other opportunities reported included: educating people including policy makers, academics and health professionals; introducing supporting compassion across services and sectors; upskilling the local workforce in remote or small jurisdictions instead of attracting workforce from other areas; and building on existing services as illustrated by quotes such as:

Our health professionals are not educated specifically around these areas, and I’m talking about psychiatrists and psychologists, GPs, clinicians, a whole range of clinical workforce members, but also non-clinical workforce members;

Focus on residential workforce rather than incentivising people to come from interstate and join our workforces; and.

Building on what we know works and introducing better services rather than dividing services and reinventing the wheel.

## Discussion

This study elicited key informant perspectives of successes, challenges, and opportunities in government-led suicide prevention efforts in Australia. Key successes included: leadership and funding for programs, services, and research; valuing the collective lived experience voice; moving towards a whole-of-government/system approach; and high community and political suicide (prevention) awareness. Key sector challenges included: defining the suicide prevention sector, limitations in the service system, workforce issues, and building the evidence base. A range of opportunities, which appeared to address some of the challenges, were mentioned for improving suicide prevention efforts, such as: leveraging the current unprecedented awareness and desire for collaboration to improve collaboration among multiple stakeholder groups; adopting wellness rather than crisis-driven models of care; including lived experience and co-design in all stages and aspects of policy planning, service development and evaluation; and investing in data, research, and evaluation.

Key stakeholder views supported the findings of a broader environmental scan of Australia’s government-led suicide prevention activity [14]. These findings and those of the broader environmental scan have implications for the development of Australia’s new National Suicide Prevention Strategy and may be relevant for suicide prevention policy and program development in other high-income countries.

For example, there is an opportunity for the new Strategy to define the scope of suicide prevention in Australia in a way that aligns with the public health framework articulated by Pirkis and colleagues (2023) [10]. Because this framework includes social determinants (macroeconomic, public and social policies; legislative/regulatory frameworks; cultural and societal values; health care coverage and system capacity and responsiveness; social cohesion and social capital) and individual risk factors (sociodemographic and other risk factors), it has potential to promote a whole-of-government and cross-sectoral approach that includes wellness models of care. The NSPO is also working on the development of a National Suicide Prevention Outcomes Framework that will also contribute to a shared understanding of intervention outcomes.

Although Australian suicide prevention policy recognises the need for a whole-of-government, cross-sectoral approach, key informants mentioned limited examples of this approach operating in practice (e.g., cross-portfolio co-commissioning services in one jurisdiction and a cross-domain policy working group in another). Therefore, a whole-of-government, cross-sectoral approach needs to be more widely and routinely implemented, embedded and evaluated in practice. The new Strategy can help foster such an approach by including explicit guidance on mechanisms for implementing and monitoring a whole-of-government, cross-sectoral approach. Cross-sector efforts can be facilitated by using shared personnel or resources, written agreements, and regular meetings [29]; or by developing a cross-government suicide prevention workplan as has been accomplished in the UK, to commit each government portfolio to taking action on suicide and outlining deliverables and timeframes for monitoring progress against commitments [30].

Although inclusion of lived experience in co-design is an area of achievement in government-led suicide prevention in Australia, there remains scope for the value of lived experience (leadership and peer-led services) to be better recognised sector wide. This key informant view is consistent with the overall environmental scan and another evidence review of co-creation practices in suicide prevention in government policy [14, 31]. The latter found that Australian governments support collaboration with people with lived experience and other stakeholders through partnership and co-design, but this can be strengthened by applying other concepts such as co-creation, co-ideation, co-implementation, and co-evaluation [31]. Lived experience participation needs to be extended from the levels of consultation and collaboration to empowerment (i.e., having final decision-making power) [10]. There is an opportunity for the new Strategy to articulate a systematic approach to lived experience inclusion and include this work in the monitoring and reporting on the Strategy [32].

Key informants identified successes, challenges and opportunities related to data. The collection, collation, accessibility, and reporting of suicide related data in Australia has improved because of the establishment of the National Suicide and Self-Harm Monitoring System (NSSHMS), which includes ambulance attendances data, intentional self-harm hospitalisations data, and suicide registers based on coroners’ data in five of Australia’s eight jurisdictions [33]. Policy perspectives suggest that the suicide prevention evidence base in Australia could be further bolstered by: establishing registers in the remaining jurisdictions (currently underway); improving the availability of real-time data on suicide and self-harm; routinely utilising program logic approaches in evaluations of interventions to identify program outputs/outcomes and system outcomes; maximising data linkage opportunities that enable investigation of the effects of interventions that target social determinants of suicide; focusing efforts on collecting suicide-related data from priority populations; conducting research with input from people with lived experience and evaluating the impact of doing so; and optimising timely translation of research in policy and practice which is a focus of the LIFEWAYS project [21, 34].

Finally, ongoing improvement of Australia’s suicide prevention efforts will require commensurate improvement in resourcing, leadership and coordination. Governance structures and accountability measures are critically needed to help all government tiers, jurisdictions, portfolios, and agencies and services work together to set priorities, define roles and responsibilities, allocate funding and report on agreed outcomes [35]. Additionally, the capacity and capability of the clinical and non-clinical suicide prevention workforce needs to be strengthened and integrated. The NSPO is planning to develop a world-first National Suicide Prevention Workforce Strategy [36], which will consider the current challenges, making an invaluable contribution to the sector’s strength.

### Limitations and strengths

Limitations of this study are that it focussed on the views of leaders involved in government-led suicide prevention policy and research and peak bodies. Therefore, the views of other (non-health) government portfolios and the community not-for-profit sector are not represented. However, we ensured representation of key sector stakeholders and their views supported the findings of the overall environmental scan, which included a review of key policy documents and a scan of programs and services [14].

## Conclusions

Achievements in Australia’s suicide prevention efforts comprise leadership and funding for programs, services, and research; valuing the collective lived experience voice; shifting towards a whole-of-government/system approach; and high community and political suicide (prevention) awareness. Suicide prevention in Australia is hampered by unclear delineation of the suicide prevention sector, limitations in the service system, workforce issues, and shortcomings in building the evidence base. Opportunities for improvement include: leveraging the current unprecedented awareness and desire for collaboration among multiple stakeholder groups; adopting wellness rather than crisis-driven models of care; including lived experience and co-design in all stages and aspects of policy planning, service development and evaluation; and investing in data, research, and evaluation. Ongoing concerted efforts are needed to define and build the capacity of the suicide prevention sector, implement and monitor a whole-of-government approach that includes wellness models of care and lived experience, and bolster the evidence base. These efforts require effective leadership and ongoing resourcing. Australia’s new Strategy will require support and resources for implementation and its effectiveness and cost-effectiveness should be evaluated.

## Data Availability

No datasets were generated or analysed during the current study.

## Abbreviations

COREQ: Consolidated Criteria for Reporting Qualitative research
NACCHO: National Aboriginal Community Controlled Health Organisation
NSPLSP: National Suicide Prevention and Leadership Support Program
NSPO: National Suicide Prevention Office
NSSHMS: National Suicide and Self-Harm Monitoring System
LGBTIQ+: Lesbian, Gay, Bisexual, Transgender, Intersex, Queer/Questioning
PHN: Primary Health Network
